# Are South African Speech-Language Therapists adequately equipped to assess English Additional Language (EAL) speakers who are from an indigenous linguistic and cultural background? A profile and exploration of the current situation

**DOI:** 10.4102/sajcd.v63i1.130

**Published:** 2016-03-18

**Authors:** Thandeka Mdlalo, Penelope Flack, Robin Joubert

**Affiliations:** 1 Speech Therapist, Livingstone School, Durban, South Africa; 2 Discipline of Speech Language Pathology, University of KwaZulu-Natal, South Africa; 3 Discipline of Occupational Therapy, University of KwaZulu-Natal, South Africa

## Abstract

This article presents the results of a survey conducted on Speech-Language Therapists (SLTs) regarding current practices in the assessment of English Additional Language (EAL) speakers in South Africa. It forms part of the rationale for a broader (PhD) study that critiques the use of assessment instruments on EAL speakers from an indigenous linguistic and cultural background. This article discusses an aspect of the broader research and presents the background, method, findings, discussion and implications of the survey. The results of this survey highlight the challenges of human and material resources to, and the dominance of English in, the profession in South Africa. The findings contribute to understanding critical factors for acquiring reliable and valid assessment results with diverse populations, particularly the implications from a cultural and linguistic perspective.

## Background to the research survey

This article presents an aspect of a larger PhD study, the aim of which was to critically evaluate and interrogate the use of language assessment tools, in their current form, within the South African context and to produce guidelines for adaptations to these tools that will better accommodate English Additional Language (EAL) speakers. These guidelines and principles could be used by language professionals to manage the assessment process and interpretation of findings from EAL speakers in a more accurate, appropriate and equitable manner. The term ‘EAL speaker’ is used here to specifically refer to South African multilinguals who are non-mother tongue speakers of English and are from indigenous language and cultural backgrounds^1^.

The larger study makes use of a specific screening tool as a model, to exemplify and illustrate the argument. In this study, the cultural and linguistic relevance of this commonly used screening tool is interrogated from four different viewpoints: firstly, the perspective of the children, who are the target population of the tool; secondly, that of the parents and community, who play a significant role in the socialisation of the children; thirdly, from the perspective of the academics from an indigenous language and cultural background, who provide an academic perspective of the tool; and, finally, that of Speech-Language Therapist (SLT) practitioners who administer the tool and interpret the findings. As language assessments are conducted on people who exist within a cultural context, the cultural capital is embedded in language (Peltier, 2010; Seidman, 2008; Westby, 2009). Since the larger study focuses on the relationship between language and culture and adopts an ecological approach to the problems addressed in the study, a conceptual model that encompasses a strong ecological and cultural component was selected; that is Taylor’s (1986) cultural framework for viewing normal and pathological communication.

As part of this larger study, a national survey was conducted with SLTs to establish current practices in assessment and intervention, training and challenges experienced when working with a client who is an EAL speaker.

The discipline of Speech Language Pathology (SLP) has a clientele, both in South Africa and globally, which is becoming increasingly multilingual and multicultural (Jordaan, 2008; Williams & McLeod, 2012; Wium, 2010). The assessment of diverse populations needs to take into account and to accommodate this diversity of languages and cultures (Gopaul-McNicol & Armour-Thomas, 2002; McLeod, 2014; Tabors, 2008). The provision of a culture-fair assessment, however, presents many challenges to the profession (Caesar & Kohler, 2007; Landsberg, 2005; McLeod, Verdon & Bowen, 2013; Pillay, 2003). These include the limited knowledge and understanding that therapists have of the cultures and language groups from which their clientele may come. Knowledge of the language and cultural background of clients is significant for the therapist as it influences the outcome of assessment and interpretation of their findings, especially when the therapist is likely to use their own worldview as a basis for this process. It also aids the SLT in discriminating between a language disorder and language difference in EAL speakers (N. Miller, 1984; Thordardottir, 2011).

Language pathology may include: difficulty in understanding or expression of the meaning of language, problems with understanding or appropriate use of the grammatical or morphological (involving units of meaning) rules, appropriate use in social context and problems with speech sounds, patterns or rules of organisation (Shipley & McAfee, 2004). The difficulties described here manifest in whatever language the individual uses. It is thus a language problem and not a second language (L2) difficulty. On the other hand, there may be language differences in the production by the individual, in the process of learning a L2 (i.e. undergoing bilingualism). These differences, which may be perceived as errors by a non-informed ear, will only manifest in the L2 or language being developed rather than the mother tongue. They may be influenced by factors such as language, culture and frame of reference.

Appropriate training plays a crucial role in facilitating increased linguistic and cultural understanding of the client’s background (Du Plessis, 2010; Higgs, 2010). As these challenges are relevant for the South African context there is a need for research to address the gap in the assessment of our diverse population. This is the rationale for the survey, the outcome of which is discussed in this article.

## The research method

The broader PhD study used a mixed methods approach with multiple data collection methods such as a survey, focus groups, individual interviews, test administration and consensus methods. The methodological design was comprised of two phases with the national survey being part of the preparatory phase, in order to set the foundation for and support the rationale for the research. Apart from establishing the assessment and therapeutic interventions that SLTs use with their clientele, the survey also assisted in providing an indication of the profile of the South African SLT.

A national survey of 1000 SLTs, registered with the Health Professions Council of South Africa (HPCSA), was thus conducted using random sampling.

Questionnaires with open and closed-ended questions on areas related to employment, clients, caseload, choice of language for practice, current practices in assessment and intervention, training and challenges were sent to SLTs on the register. Firstly, a pilot study was conducted by sending 100 questionnaires to SLTs (10%) from this national register and the questionnaire was revised, taking into account these responses. Based on the responses received, changes were made, such as rephrasing of ambiguous questions on language use and current management and extension of some of the options provided in questions on caseload, employment and experience with EALs.

The survey questionnaires were then sent to 1000 HPCSA registered SLTs via the postal service and a 15% (∑150) response rate was achieved. Despite the relatively low response rate, the results are consistent with findings of global and local research (Jordaan, 2008; Von Dulm & Southwood, 2013), which had similar response rates. Data were analysed using the Statistical Package for Social Sciences (SPSS 18). The data were organised into simple frequencies and presented in tables and graphs.

## Results of the survey

**FIGURE 1:**
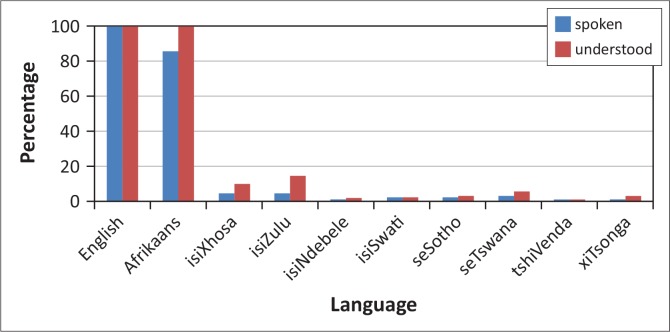
A bar chart showing the language competence percentages of SLTs in SA (Data to be interpreted with caution due to low response rate).

The results of the survey show that 99% of SLTs sampled were from English or Afrikaans speaking backgrounds and competent in these languages (Figure 1). Furthermore, 89% of these SLTs had EAL speakers in their caseload and 86% of the SLTs used English in the assessment of these EALs. The results also suggest that English standardised language assessment tools, which have been normed on populations predominantly in the US and UK and are inappropriate for EAL speakers, remain the tests most commonly used by SLTs to assess this population and they are administered in English.

There are several reasons given by the therapists for the choice of English in assessments, but the most common is the therapist’s self-proclaimed restricted competence in other African languages, as reflected in the quotes below:

‘I feel equipped when the goal is to improve English language comprehension and expression’.

‘I only work in English medium schools where I have an understanding of culture or background and thus sensitivity thereof’.

‘I feel equipped because I don’t take on children if therapy is not in English’.

‘My level of competence in understanding, speaking and thinking about all languages (except English) is insufficient in providing quality accountable service’.

Although some of the therapists are content with the use of English, as reflected in their responses, others expressed some concerns:

‘Assessment tools and programmes are foreign to these children (referring to EAL speaking children) regarding the language as well as cultural barriers’.

‘There are several reasons why these assessment tools present with these barriers and these include a different language, culture, experience or dialect’.

## Discussion and implications of the results

Because of the low response rate to the questionnaires, the findings should be viewed with some caution in terms of conclusiveness. They do however provide an interesting insight into the direction of a broader picture of the profile of SLTs and their test usage in SA.

The survey findings suggest that EAL speakers are currently mostly being assessed by SLTs who come from different linguistic and cultural backgrounds to those of the clients (SASLHA, 2012, 2013; Von Dulm & Southwood, 2013). This information accentuates the issue of the relationship between language and culture. Language represents a powerful tool of self-definition and expression and becomes a means through which various cultural and social groups can find unique expression (Han & Price, 2015, Jandt, 2000; Ji, Zhang & Nisbett, 2004; Tabors, 2008). It can thus be argued that language is a cultural phenomenon (Ball & Peltier, 2011; Riley, 2007; Sardar & Van Loon, 2004). Bearing this in mind, it becomes essential for professionals in the language field to understand and always draw on mother tongue, socio-cultural meanings when involved in the assessment of language of multilingual and multicultural populations (Gopaul-McNicol & Armour-Thomas, 2002; Solarsh & Alant, 2006; Westby, 2009). In South Africa, the majority of EAL speakers use an African language as their mother tongue (http://www.statssa.gov.za), and the majority of SLTs do not. The survey results indicate that EAL speakers are thus currently being evaluated by SLTs who do not speak or understand an African language. In addition, feedback on the questionnaire also reveals that SLTs have restricted understanding of the cultures linked to these languages. It can thus be assumed that the SLT will use their own linguistic and cultural background as a frame of reference for interpreting the assessment results and this in turn may further influence the language choice and use in assessment as well as interpretation of results.

The picture thus emerging from the results of the survey places the SLT in a very powerful position as they can attach their own cultural and linguistic worldview to the meaning of the assessment and criteria for success. This powerful position further raises the question as to whether the meaning that the SLT attaches to their assessment, serves the interests of justice and equality as they relate to the client. Issues of justice and equality underpin current discussions within the profession pertaining to hegemonic discourses, language and practices that reproduce them (Kathard & Pillay, 2013, 2015).

The nature and number of comments from some of the respondents suggest that they concur that the current situation is not ideal and indicate the need for a greater research effort into the creation of more culturally and linguistically relevant language assessment materials for EAL speakers in South Africa.

As a result of the challenges mentioned by the SLTs in the survey, specifically a paucity of culturally and linguistically relevant tests developed for the South African population (Naudé, Louw & Weideman, 2007; Pascoe & Norman, 2011), most evaluations are conducted using tests developed and normed on populations that are predominantly from the US or UK. That these tests are not ideal in their current form for assessing a South African EAL speaker who is from an indigenous language and cultural background goes without saying and this is confirmed by research into language development and assessment (Caesar & Kohler, 2007; Gopaul-McNicol & Armour-Thomas, 2002; Moro, 2008; Pierce & Williams, 2013). Tests currently used by SLTs in South Africa are predominantly based on a linguistic, cultural and social context that is largely European and American. The guidelines for the administration of these tests do not refer to the South African multilingual and multicultural population. The test developers thus do not expect them to be used on populations outside of those stipulated in the test.

One of the reasons that the SLTs provided for mostly conducting the assessment and therapy of EAL speakers in English is the demand for the use of English by the parents of the EAL speaking children. These demands are linked to the perception by parents of EAL children that English is the language linked to progress in education. Despite the recognition of 11 official languages in SA, English remains the dominant language in all sectors of society (Burger, 2011; Green, 2008; Kamwangamalu, 2000; Landsberg, 2005; Muendane, 2006), including education (Republic of South Africa, 2011). Many African EAL speaking children from an indigenous language and cultural background are thus taught in settings where English is the medium of instruction (MoI). The language of learning and teaching (LOLT) in many South African schools is English (Lafon, 2007; Landsberg, 2005). Many SLTs therefore try to justify their persistence in assessment and intervention in English even for EAL speakers, the evidence of which is clear in the survey results, which showed that 86% of the SLTs used English in language assessments and therapy.

The results of this survey are particularly crucial, because of the many EAL speakers from indigenous cultural and linguistic backgrounds in South Africa who are in schools where English is the MoI and who are referred by teachers for language assessments to SLTs. Even though the curriculum may be presented in English, this is not the mother tongue of these South African children, nor is their cultural background reflected (Higgs, 2010; Landsberg, 2005; Ntuli, 2002).

African language and culture influences the knowledge and belief system of the EAL speaker (Lemmer, Meier & Van Wyk, 2006; Metz & Gaie, 2010) and therefore influences their response to the tests, which have been predominantly normed on US or UK populations, societies whose language and culture is different from theirs (Higgs, 2003; Kroes, 2005; Makgoba, 1999; Semali, 1999). Therefore, the background, values and stories of the EAL speaker, such as found in traditional oral African culture manifesting in African signs and symbols (Maathai, 2009; Mutwa, 1998), tend to be disregarded, devalued, ignored or only superficially addressed (D. Miller, 2012) when resources for evaluations do not reflect the African experience.

As the majority of EAL speakers in South Africa are African language mother tongue speakers, it can be said that the child is treated as a tabula rasa when the African worldview (Behrens, 2010; Mucina 2013; Ntuli, 1999, 2002) they bring is ignored. The children’s choice, based on their frame of reference, is excluded as an option in the assessment tool scoring system. The outcome may likely thus create a distorted reflection of the language ability of an EAL speaker from an indigenous language and cultural background and the SLT may thus, unintentionally, pathologise a child who presents with a language difference.

## Conclusion

The results of the study indicate that the average SLT assessing an EAL child is still predominantly either an English or Afrikaans speaking woman, who is not competent in an African language. Saville-Troike (1986, p. 48) maintains that ‘whether we realise it or not, each of us sees the world from a culturally conditioned perspective that we share with the other members of the group’. Thus, the frame of reference of these SLTs is based upon their own socio-cultural background, which in turn influences their interpretation of the child’s response. These findings have implications for the selection and training of SLTs in South Africa and suggest a direction for postgraduate research in this discipline. The current recruitment of African language speakers for training in the profession needs to be intensified to accommodate the assessment and management of this population. Sections of the Speech-Language Pathology curriculum pertaining to bilingualism and cultural and linguistic diversity need to be enhanced for more effective preparation of the SLTs who will work with EAL speakers. In addition, postgraduate research that addresses these discrepancies should be encouraged.

Although it is necessary that further research be conducted to create more culturally and linguistically relevant tools for the EAL population, the practising SLTs remain accountable for accessing research-based evidence on the assessment and management of the EAL population. Failure to do so constitutes a contravention of the profession’s ethical code of conduct. It is hoped that the outcome of this research will create greater sensitivity in the application of non-standardised language screening tests to EAL speakers in this country.

## Footnotes

1 Not a homogenous group there may be inter- and intra-linguistic and cultural variability
